# Research on a High-Efficiency Goat Individual Recognition Method Based on Machine Vision

**DOI:** 10.3390/ani14233509

**Published:** 2024-12-04

**Authors:** Yi Xue, Weiwei Wang, Mei Fang, Zhiming Guo, Keke Ning, Kui Wang

**Affiliations:** 1 Research Centre for Intelligent Farming Equipment, Anhui Agricultural University, School of Engineering, Anhui Agricultural University, Hefei 230036, China; xueyi123@stu.ahau.edu.cn (Y.X.); wwwang@ahau.edu.cn (W.W.); guozhiming@stu.ahau.edu.cn (Z.G.); gxydd@stu.ahau.edu.cn (K.N.); 2 College of Mechanical Engineering, Anhui Science and Technology University, Chuzhou 233100, China; fangm@ahstu.edu.cn

**Keywords:** precision farming, machine vision, multi-source fusion, identity recognition, multi-view appearance, decision fusion

## Abstract

Accurate identification of individual goat identity is necessary for precision farming. Previous studies have primarily focused on using front face images for goat identification, leaving the potential of other appearances and multi-source appearance fusion unexplored. In this study, we used a self-developed multi-view appearance image acquisition platform to capture five different appearances (left face, right face, front face, back body, and side body) from 54 Wanlin white goats. The recognition ability of different goat appearance images and its multi-source appearance fusion for its identity recognition was then systematically examined based on the four basic network models, namely, MobileNetV3, MobileViT, ResNet18, and VGG16, and the best combination of goat appearance and network was screened. When only one kind of goat appearance image was used, the combination of side body image and MobileViT was the best, with an accuracy of 99.63%; under identity recognition based on multi-source image appearance fusion, all recognition models after outlook fusion of two viewpoints generally outperformed single viewpoint appearance identity recognition models in recognizing the identity of individual goats; when three or more kinds of goat appearance images were utilized for fusion, any of the four models were capable of identifying the identity of an individual goat with 100% accuracy. Based on these results, a goat individual identity recognition strategy was proposed that balances accuracy, computation, and time, providing new ideas for goat individual identity recognition in complex farming contexts.

## Simple Summary

Precision animal farming is premised on individual identity recognition, and most existing research use images of front faces to recognize the individual identity of goats because of the inertia of human face recognition. However, due to the rapidity of goat head movement and the high convex of the face structure, a qualified front face image is often not easy to capture under natural conditions. Finding appearances that are easier to capture and more accurate in identifying individual goat identities is valuable for practical precision production. This study revealed the ability of the 54 goats’ five outward appearances and their fusion to mark their individual identity. The results showed the side body image provided the highest accuracy, followed by the back body and front face images, with minimal differences between the side face views. The highest accuracy was achieved with the side body image using MobileViT when only one appearance image was used. Two appearances generally outperform one appearance in recognition accuracy; when three or more appearances were utilized for fusion, any of the models have an accuracy of 100%. These findings form a solid foundation for developing practical goat identification systems.

## 1. Introduction

Precision farming is increasingly essential for reducing costs and increasing farm efficiency [1], with the scale of goat farms constantly expanding [2]. Accurate identification of a goat’s identity is indispensable for efficient farm management, vaccination, disease prevention, selection of superior breeding stock, mortgage loans, product traceability, etc. [3].

Traditional identification methods, such as ear cutting, hot iron branding, and tagging with ear tags [4], cause permanent physical and mental damage to domestic animals [5]. Current radio frequency identification (RFID) technology [6] is relatively expensive, susceptible to interference by ferrous products, could be artificially tampered with [7], and cannot be applied over long distances [8]. Machine vision technology can rapidly and accurately recognize animals’ identity through their biological features such as nose prints [9], iris patterns [10], retinal images [11], and facial characteristics [12], which are becoming a hot topic in current research [13]. Among those biological features, nose prints, iris patterns, and retinal images are challenging to collect and inconvenient for widespread application [14]. Facial features, however, can be remotely recognized without direct contact with the livestock, offering advantages such as naturalness, non-contact, non-invasiveness, and ease of collection [5], attracting increasing attention in recent years. For example, Hansen et al. captured facial images of pigs through a camera near a drinking facility and used a convolutional neural network (CNN) to recognize the individual identity of pigs corresponding to the face images with an accuracy of 96.7% [15]. Salama et al. used the CNN model optimized by the Bayesian network to recognize the identity of 52 sheep faces with significant age differences with 98% accuracy [16]. Gong et al. took cow face images from different angles to build a cow face dataset and proposed an SK-ResNet recognition model with 98.42% accuracy [17]. Billah et al. first detected goats’ face, eye, nose, and ear features by the YOLOv4 algorithm. Then they recognized the identity of cropped and aligned goat face images using a custom CNN model with 96.4% accuracy [18]. Hitelman et al. used the camera on the self-service watering device to record videos of sheep while they were drinking [19]. Then, the sheep face image in the videos is detected through the Faster R-CNN model, and the identity of sheep face images is recognized by the ResNet50V2 model with the ArcFace loss function, with an average accuracy of 97%. Li et al. proposed a lightweight MobileViTFace model for sheep face recognition with 97.13% accuracy for 186 sheep identities [20].

Previous studies have shown [5,16,19] that the individual identity of the animal can be recognized using appearance images, and the front face image seems to be the best choice. However, whether the front-face image is the best choice for recognizing goat identity like other livestock has not been confirmed until now. In fact, compared with other livestock, the goat’s face is covered with more wool and is more convex; therefore, the information on the front-face appearance may not be as rich as that of other animals. It is important to reveal whether the front face of a goat is the one with the best performance for identity recognition and to explore which appearance performs best for identity recognition. On the other hand, it is inefficient and difficult to identify a goat using only the front-face appearance. This is because goats are naturally active and have diverse head postures, and it is tough to obtain an ideal front-face image of a goat under unconstrained conditions [18]. Zhang et al. confirmed that using multiple facial images could more accurately identify sheep, but did not further explore the potential of sheep’s other appearance images [21]. To the best of our knowledge, no study systematically compares the ability of left side view of the goat face, front view of the goat face, right side view of the goat face, top view of the goat back body, and side view of the goat body on goat individual identity recognition, let alone exploring the performance of the fusion of different view appearance image, which hinders the establishment of more practical and accurate methods of identifying individual goat.

Based on the above considerations, this study created a set of goat multi-view appearance image acquisition platforms, and the left side view of the goat’s face, front view of the goat’s face, right side view of the goat’s face, top view of the goat’s body, and side view of the goat’s body were collected from 54 Wanlin white goats. The ability of the goat’s different appearances and their fusion on goat identity identification were systematically investigated based on the four basic network models, MobileNetV3, MobileViT, ResNet18, and VGG16, and we screened the optimal combination of goat appearance and network.

The main contributions are as follows:

(1) We propose a set of multi-view appearance image acquisition platforms, providing a new idea for the multi-view appearance image acquisition of goats under natural feeding conditions.
(2) The performance of different goat appearance images and high-efficiency multi-source appearance fusion utilizing appearance from different viewpoints on individual recognition was revealed, providing theoretical support for the research and development of goat individual recognition systems.
(3) We proposed a method for recognizing individual goats based on multiple external appearances, which provides solid technical support for the popularization of the application.

Based on the achievements of this study, livestock farming managers can quantitatively trade-off between identification accuracy, identification computation, and hardware cost to choose the identification method that belongs to the required one. Such outcomes provide solid theoretical support for promoting machine-vision-based sheep individual identification technology to serve precision livestock production.

## 2. Materials and Methods

### 2.1. Experimental Overview

**Figure 1:**
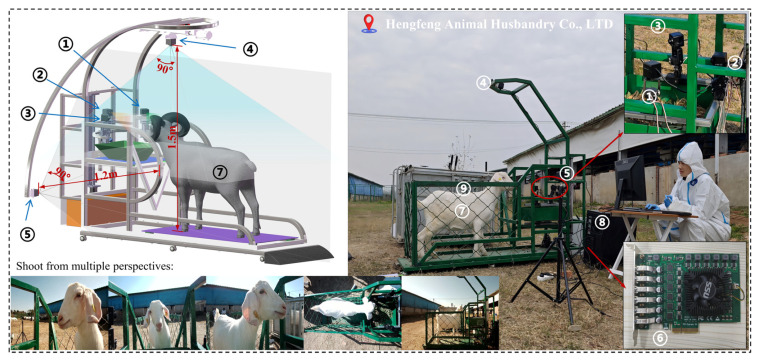
Scene diagram for collecting a goat’s five appearance images. Here, “1” is the camera capturing the right face; “2” is the camera capturing the front face; “3” is the camera capturing the left face; “4” is the camera capturing the back body; “5” is the camera capturing the side body; “6” is a PCI-E to USB 3.0 expansion card (6 channels); “7” is the goat under test acquisition; “8” is the desktop computer; “9” is the restraining frame.

The experiment of this study was approved by the Experimental Animal Welfare Ethics Committee of Anhui Agricultural University (Animal welfare ethical number: AHAUXMSQ2023123). The experiment was conducted from November 11th to 25th, 2023, at Hengfeng Animal Husbandry Co., Ltd., in Yuanzhuang Village, Zhangguan Town, Linquan County, Fuyang City, Anhui Province, China (32°54′12″ N, 115°18′40″ E). A total of 80 Wanlin white goats with very similar appearance of 6~8 months of age, with an average weight of approximately 30 kg, were employed. Five types of appearance images of every goat were captured using the platform shown in Figure 1: left face image, front face image, right face image, back body image, and side body image. Considering the effect of light intensity, this experiment was conducted at different times of the day (8:00~10:00, 13:00~15:00, 16:00~18:00).

The acquisition platform mainly consists of a restraining frame, a feeding device, 5 RGB cameras, and a desktop computer. The RGB cameras are of the Sony IMX335-USB3.0 brand and have a resolution of 5 MegaPixels, with a horizontal shooting angle range of approximately 90°. They were connected to the desktop computer via a PCI-E to USB 3.0 expansion card (6 channels), and the VideoCap (version 1.27.0.22) software was employed to record videos of the goat’s appearance. We captured the optimal goat appearance images by adjusting the angles of the five cameras: one camera installed directly above the restraining frame was adjusted to capture an overhead view of the goat’s body; three cameras captured the left side, front, and right side of the goat’s face, respectively, by adjusting the lifting of the acquisition bracket with a lead screw motor to the appropriate height and further remotely adjusting the angles of the two-degree-of-freedom servo gimbal; and another camera was positioned approximately 120 cm above the ground and 40 cm from the side of the restraining frame to capture a side view of the goat’s body.

### 2.2. Experimental Procedures

Before the formal experiment, each goat was rotated to enter the restraining frame twice to acclimatize them to the testing environment. No food was provided to the goats for half a day before the formal trial to keep them in a fasted state to encourage natural eating behavior. In the formal experiment, each goat was induced into the restraining frame for feeding by adding palatable forage. During the feeding process, videos of the goat feeding were simultaneously recorded from five viewpoints using the VideoCap software, with the video frame rate set to 60 frames per second and the image resolution set to 1920 × 1080 and saved in AVI format. Each goat was filmed for about 5 min, and after the trial, the goat was released back into the pasture. The appearance videos of all 80 goats were captured. However, due to the overstress of some goats and the malfunction of the acquisition system, the five appearance videos of 16 goats were incomplete. Ultimately, only 54 goats had complete videos of all five appearances.

### 2.3. Dataset Creation and Preprocessing

**Figure 2:**
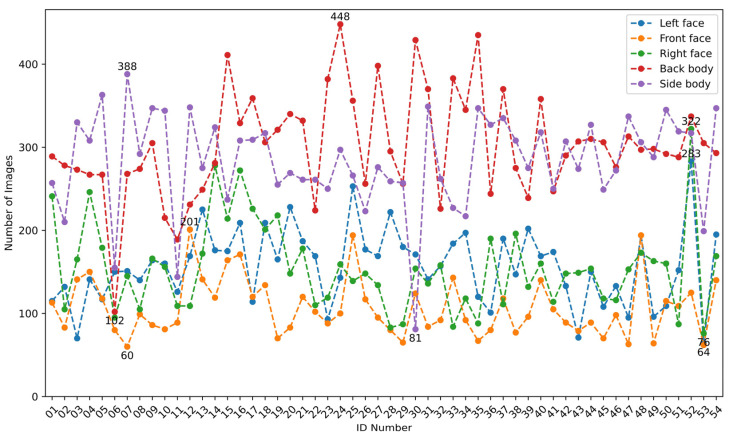
Distribution of the number of different appearance images of goats in the original training set. The horizontal axis is the ID number of the experimental goat (taking the last two characters of the number), and the vertical axis is the number of images corresponding to the different appearances of each goat ID.

**Figure 3:**
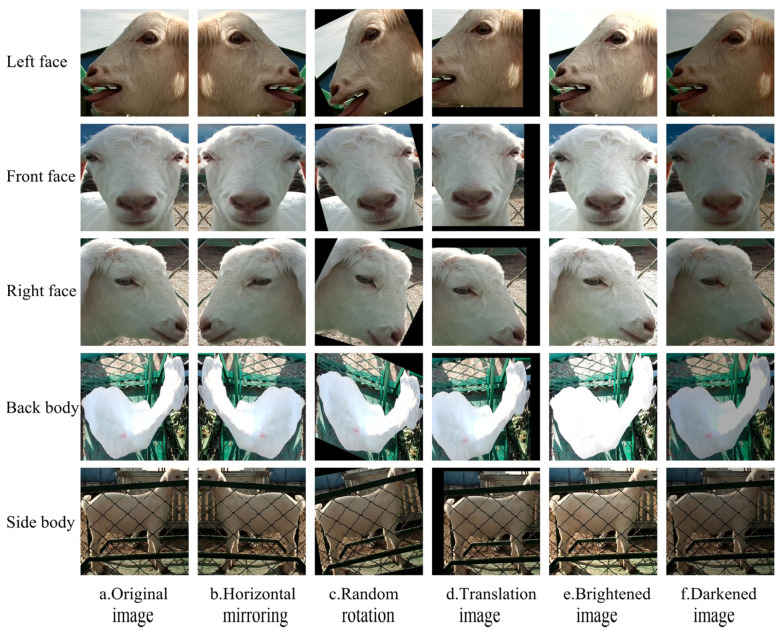
Example images of the augmentation of five external images of a goat. From left column to right column: original image, horizontal mirroring, random rotation, translation image, brightened image, darkened image.

Images were extracted at intervals of 30 frames from the videos of 80 goats, and images without goats or with blur were deleted to obtain the raw image set. Subsequently, each raw image was further annotated to indicate the area where the goat’s body or face was located. Due to manual annotation’s time-consuming and labor-intensive nature, this study utilized the YOLOv8n [22] object detection algorithm, which is capable of maintaining high-speed inference while providing high-quality detection results to assist in annotating the goat’s appearance. Subsequently, a dataset for goat individual identification was created based on the trained YOLOv8n model. It is detailed as follows:

(1) The training method of the auxiliary annotation model YOLOv8n. Firstly, appearance images of 20 goats were randomly selected from 54 data-complete goats, plus images of another 16 data-incomplete goats were used. Adjacent frame images were filtered to remove highly similar ones using the dHash perceptual image hashing algorithm (Hamming distance dist < 5), resulting in a total of 17,020 images as the dataset. Secondly, 800 images of each of the five types of goat appearance were randomly selected (4000 in total) from the dataset, and Labelimg (version 1.8.6) software was used for manual annotation. We annotated the left face, the front face, the right face, the back body, and the side body of the goat with the minimum bounding rectangle, respectively. Among them, the left face refers to a goat’s left side face with only one eye visible; the front face refers to a goat’s face clearly showing both eyes; the right face refers to a goat’s right side face with only one eye visible; the back body refers to the goat’s back body in a normal standing position on four legs; and the side body refers to the goat’s side body in a normal standing position on four legs. Finally, the YOLOv8n model was initially trained based on 4000 images manually labelled, and the trained YOLOv8n model was used to automatically annotate the other images (13,020 images). Automatically annotated images were manually reviewed and adjusted to ensure correctness. The annotated 17,020 images were divided into training and validation sets at a ratio of 9:1, and the YOLOv8n model was trained again to obtain a model capable of accurately locating the goat’s appearance area.

(2) Preprocessing and partitioning the dataset for goat individual identity recognition. This study conducted subsequent research on goat individual identity recognition based on appearance videos of 54 data-complete goats. The 54 goats were numbered from 0001 to 0054, and five types of appearances were defined as Left face (L), Front face (F), Right face (R), Back body (B), and Side body (S). For the image sequences extracted from the 54 goats, the YOLOv8n model trained above was used to automatically locate and crop the goat’s appearance area and resize the image to 224 × 224 pixels. To ensure the diversity of goat-appearance images, the Structural Similarity Index (SSIM) [23] was utilized to compare the brightness, contrast, and structure of neighboring images (the threshold was set to 0.7), and similar goat-appearance images were deleted to obtain the goat’s appearance image set. From the goat’s appearance image set, 20 images were randomly selected from each type of appearance of every goat, totaling 5400 images, to form the test set, and the remaining images were used as the training set. The distribution of the number of images for each type of goat appearance in the training set is shown in Figure 2. Among them, there were 8465 images of the left face, 5779 images of the front face, 8336 images of the right face, 16,334 images of the back body, and 5323 images of the side body, showing severe imbalance in the number of various appearance images of different goats (with the highest number of side body images for goat 0024, reaching 448; and the fewest front face images for goat 0007, only 60). To balance the number of samples in each class to avoid model bias and improve the generalization ability of the model, this study used image augmentation techniques to expand the images of each type of appearance of each goat, as shown in Figure 3. So, the number of each appearance image of each goat was expanded to 450. The augmented training set included 121,500 images, which were further randomly divided into training and validation sets at 8:2.

### 2.4. Identity Recognition Method

**Figure 4:**
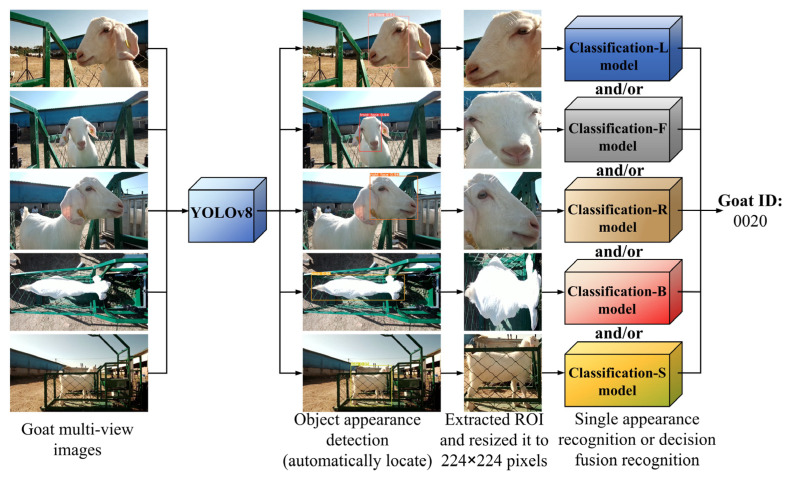
Flowchart of identifying individual identity using multiple types of goat appearance images.

In this study, the self-trained YOLOv8n model was used to automatically locate the appearance image of the goat from the image extracted from the video as the region of interest (ROI). These ROIs were extracted and resized to the currently dominant 224 × 224 pixel size and then fed into the single appearance recognition or decision fusion recognition classification model to recognize the identity of the goat individual, as shown in Figure 4.

#### 2.4.1. Base Network Model

To thoroughly reveal and compare the ability of five appearance types to identify goats’ individual identity, the four most representative backbone networks, MobileNetV3, MobileViT, ResNet18, and VGG16, were selected to construct the base network model.

MobileNetV3 [24] has demonstrated excellent performance in tasks such as image classification, object detection, and semantic segmentation, with high running speed and low resource consumption on mobile devices, making it highly promising for widespread applications. MobileViT [25], in addition to the advantages of lightweight design inherited from MobileNet, incorporates transformer self-attention mechanisms, enabling it to capture global information in images with good performance at lower model complexity and computational cost. ResNet18 [26] has relatively small-scale and efficient residual connections, exhibiting outstanding performance in training and inference, with good generalization ability suitable for various computer vision tasks, especially image classification and object detection. VGG16 [27] performed well in image classification tasks and the ImageNet Large Scale Visual Recognition Competition (ILSVRC) 2014. Despite its relatively large model size, its simple structure makes it easy to train and understand, leading to its widespread application in various computer vision tasks. In summary, MobileNetV3 and MobileViT focus on lightweight design and efficient performance on mobile devices; ResNet18 is widely adopted in practical applications due to its small model size and good generalization ability; and VGG16 is renowned for its simple and understandable structure with good performance. Each of these four models has its strengths and is representative in the field of computer vision, enabling a trustworthy evaluation of the ability of different appearances.

#### 2.4.2. Model Training Methodology

Data processing for this study was implemented on workstations running Windows 11. The workstation is equipped with an Intel Core i9-13900k 3.00 GHz CPU, 64 GB RAM, and an NVIDIA GeForce RTX 3090 GPU with 24 GB of memory. The YOLOv8n detection model and the goat identity recognition model were built and trained using CUDA 11.8, PyTorch 2.0.1 framework, and Python 3.11.

When training the YOLOv8n model, the batch size was set to 48, the worker was set to 8, the other parameters were selected as default values, and a total of 500 epochs were trained. When training the goat identity recognition model, to accelerate convergence speed and improve model accuracy, all models were pre-trained using the ImageNet dataset to obtain initial weights and then further trained on the dataset created above. Different training methods were adopted for different backbone network models. VGG16, ResNet18, and MobileViT used the SGD optimizer during the training phase, with an initial learning rate of 0.0125 and a step-wise learning rate scheduler. Specifically, the VGG16 and ResNet18 models’ learning rates decreased at the 30th, 60th, and 90th epochs, with a decay rate of 0.1. MobileViT’s learning rate decreased every two epochs, with a decay rate of 0.973. Those three models used the same momentum decay and weight decay of 0.9 and 0.0001, respectively. MobileNetV3 used the RMSprop optimizer during the training phase, with an initial learning rate of 0.001, a moving average coefficient of 0.9, and a learning rate decreased every two epochs with a decay rate of 0.973. Its momentum decay was 0.9, and the weight decay was 0.00001. All four models had a batch size of 64 during training, and the input image size was 224 × 224 pixels and were trained for 500 epochs.

#### 2.4.3. Evaluation Indicators

Goat individual identity recognition could be regarded as a classification task. Thus, the CrossEntropyLoss loss function was used to measure the difference between the model prediction results and the actual labels, and the probability that the model predicts the goat identity was output through the softmax function. The model’s overall performance was evaluated through Accuracy and the Mean Precision, Mean Recall, and Mean F1 score, as shown in Equations (1)–(7).

(1) $$\mathrm{Accuracy}=\frac{\mathrm{TP}+\mathrm{TN}}{\mathrm{TP}+\mathrm{TN}+\mathrm{FP}+\mathrm{FN}}$$

(2) $$\mathrm{Precision}=\frac{\mathrm{TP}}{\mathrm{TP}+\mathrm{FP}}$$

(3) $$\mathrm{Recall}=\frac{\mathrm{TP}}{\mathrm{TP}+\mathrm{FN}}$$

(4) $$F1\;\mathrm{score}=\frac{2\;\text{×}\;\mathrm{Precision}\;\text{×}\;\mathrm{Recall}}{\mathrm{Precision}+\mathrm{Recall}}$$

(5) $$\mathrm{Mean}\;\mathrm{Precision}=\frac{\sum_{i=1}^{N}{\mathrm{Precision}}_{i}}{N}$$

(6) $$\mathrm{Mean}\;\mathrm{Recall}=\frac{\sum_{i=1}^{N}{\mathrm{Recall}}_{i}}{N}$$

(7) $$\mathrm{Mean}\;F1\;\mathrm{score}=\frac{\sum_{i=1}^{N}{F1\;\mathrm{score}}_{i}}{N}$$

Among them, *TP* represents the true positive; *FP* represents the false positive; *FN* represents the false negative; and *TN* represents the true negative. *N* is the total number of categories; *Precision_i_* is the precision of the i-th category; *Recall_i_* is the recall of the i-th category; and *F*1 *score_i_* is the F1 score of the i-th category.

#### 2.4.4. Method of Identification

The above four basic network models were trained on each of the goat’s five appearance datasets, and a total of 20 goat single appearance identity recognition models were obtained. The performance of all models was evaluated on the test set. In every single appearance identification model, the goat appearance image is processed through the convolutional layer, pooling layer, fully connected layer and normalization to output a confidence vector of length 54 (54 being the number of goats used in this study). Each value in this confidence vector represents the probability that the model predicts the input goat appearance image to belong to a particular goat, and the index of its maximum value is the goat identity predicted by the model. The index of maximum in this probability vector, i.e., the goat identity number, can be calculated by the argmax function.

**Figure 5:**
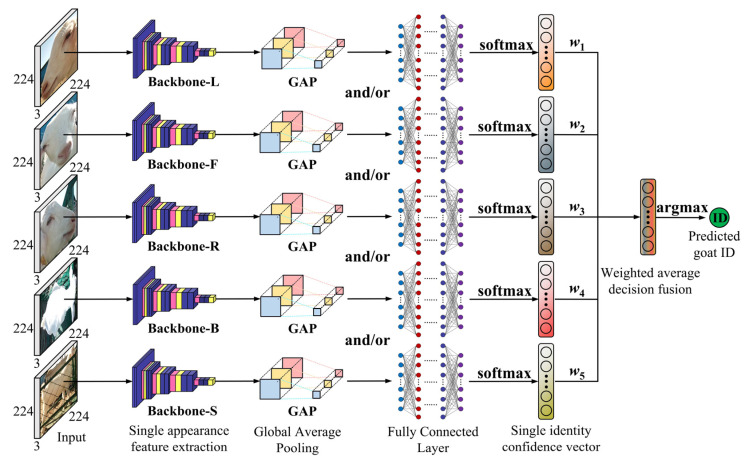
Schematic diagram of the fusion of multiple types of goat appearance images to recognize a goat’s identity.

Building on the above work, this study continued to explore the ability of fused features from two, three, four, and five types of goat appearance images at the decision level by constructing a goat identity recognition model with multi-appearance images. The multi-appearance fusion model consists of multiple branch neural networks, with each branch responsible for processing different goat appearance images. As shown in Figure 5, one branch is used to process the left side face image features; another branch is used to process the front face image features; and the other branches are used to process the right side face, back body, and side body image features. Each branch network processes the input goat appearance image independently, and its feature map (or feature tensor) is converted into a fixed-length feature vector through global average pooling and then passed into the fully connected layer for further processing. The output of the fully connected layer was processed by a softmax function to obtain the confidence vector (length 54, each data value is in the interval [0, 1]). The confidence vector of each type of appearance image is multiplied by the accuracy of the single appearance model whose appearance corresponds (i.e., the accuracy value metrics from the corresponding base model above) to obtain the weight vector. The average value of the five weight vectors is calculated to be the predicted probability vector for the identity of the goat individual under the fusion of the five appearances (the length is 54), as shown in Equation (8). The index of the largest value in this probability vector corresponds to the goat’s identity number predicted by the model. The above fusion model can utilize different goat appearance information by multiple branch networks, which theoretically performs better.

(8) $$v_{\mathrm{avg}}=\frac{\sum_{i=1}^{n}w_{i}\text{·}v_{i}}{\sum_{i=1}^{n}w_{i}}$$

where ***v****_i_* is the confidence vector corresponding to the different appearance features of the goat; *w_i_* is the accuracy of the single model corresponding to the goat’s appearance type; and ***v****_avg_* is the probability vector obtained after the weighted average.

## 3. Results

### 3.1. YOLOv8n Model

The YOLOv8n used in this study automatically locates different appearance regions of goats, and its automatic localization capability was verified by an independent test set. The detection results are shown in Table 1. The results indicate that the YOLOv8n model has very strong detection capabilities, with very high precision and recall and only a small number of missed detections or false detections. The model is highly reliable in locating objects and continues to perform excellently under strict evaluation standards (mAP@0.5–0.95).

### 3.2. Single Appearance Recognition Model

**Figure 6:**
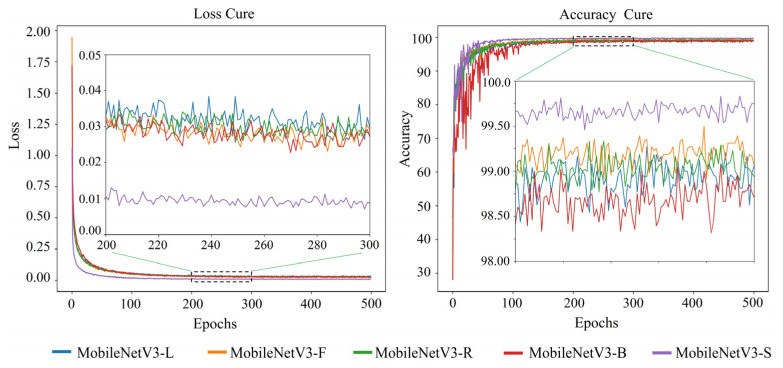
Loss and accuracy variation curves of a goat single appearance recognition model based on MobileNetV3. Here, MobileNetV3-L: model trained based on goats’ left side face images; MobileNetV3-F: model trained based on goats’ front face images; MobileNetV3-R: model trained based on goats’ right side face images; MobileNetV3-B: model trained based on goats’ back body images; MobileNetV3-S: model trained based on goats’ side body images.

**Figure 7:**
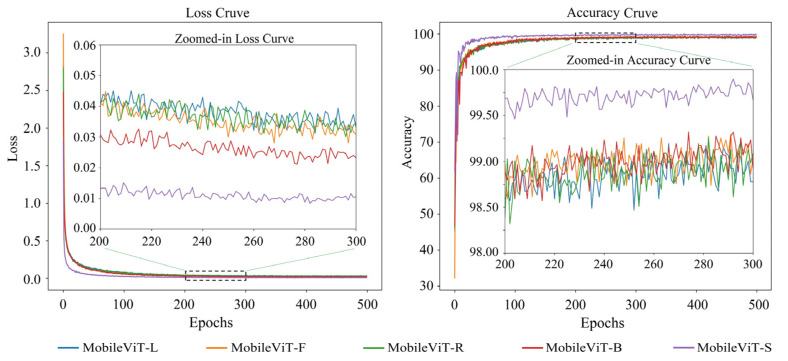
Loss and accuracy variation curves of a goat single appearance recognition model based on MobileViT. Here, MobileViT-L: model trained based on goats’ left side face images; MobileViT-F: model trained based on goats’ front face images; MobileViT-R: model trained based on goats’ right side face images; MobileViT-B: model trained based on goats’ back body images; MobileViT-S: model trained based on goats’ side body images.

**Figure 8:**
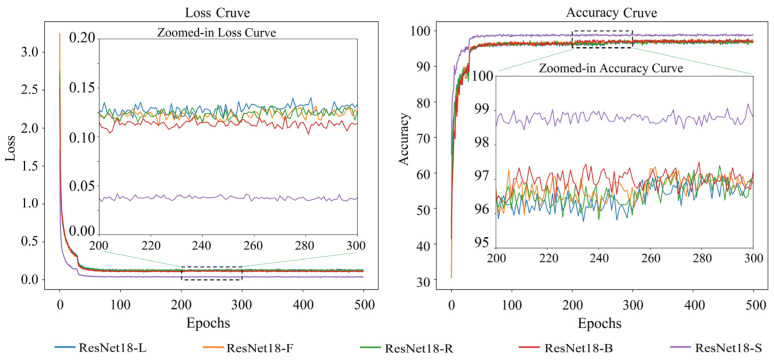
Loss and accuracy variation curves of a single goat appearance recognition model based on ResNet18. Here, ResNet18-L: model trained based on goats’ left side face images; ResNet18-F: model trained based on goats’ front face images; ResNet18-R: model trained based on goats’ right side face images; ResNet18-B: model trained on goats’ back body images; ResNet18-S: model trained on goats’ side body images.

**Figure 9:**
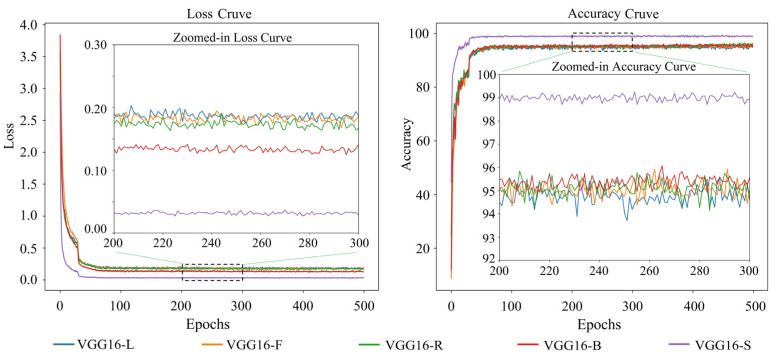
Loss and accuracy variation curves of a goat single appearance recognition model based on VGG16. Here, VGG16-L: model trained based on goats’ left side face images; VGG16-F: model trained based on goats’ front face images; VGG16-R: model trained based on goats’ right side face images; VGG16-B: model trained based on goats’ back body images; VGG16-S: model trained based on goats’ side body images.

The loss and accuracy variation curves of 20 single appearance recognition models by combining four base network models and five types of appearance images of goats during the training process are shown in Figure 6, Figure 7, Figure 8 and Figure 9.

Observation of Figure 6, Figure 7, Figure 8 and Figure 9 shows that when the base network model is the same, the decrease rate in the loss value of the model obtained from the goats’ side body appearance images is higher than that of the model of the other appearances, and the convergence rate of the accuracy value is the fastest with the highest value; the difference in the accuracy of the models corresponding to the other four appearances is not significant. Except for the side body appearance, no significant differences exist in the models corresponding to the other four appearance types based on MobileNetV3. In the models based on MobileViT, ResNet18, and VGG16, the loss of the model corresponding to the back body appearance type was slightly lower than that of the other three types of appearance.

**Figure 10:**
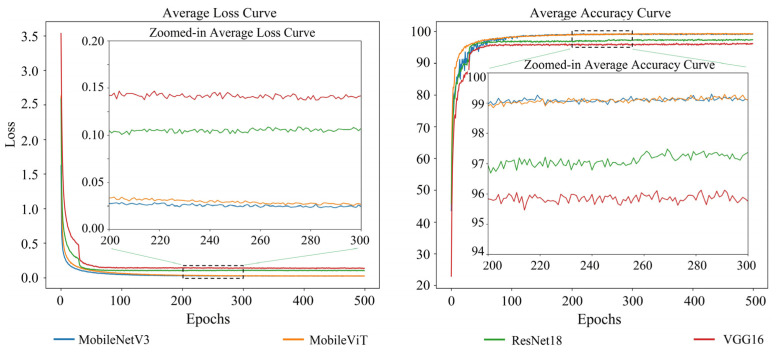
The plot of the average loss and average accuracy change curves for all view appearances for different base network models.

The average loss and average accuracy change curves for all view appearances and different base network models were calculated to compare the performance of different base network models, as shown in Figure 10. MobileNetV3 and MobileViT were not significantly different in training performance. Still, they significantly outperformed ResNet18 and VGG16, and ResNet18 was markedly better than VGG16.

The performance of 20 goats’ single appearance recognition models on the test set is shown in Table 2. Among them, the MobileViT-S model had the highest accuracy of 99.63%, and the VGG16-L model had the lowest accuracy of 93.89%. In terms of the base network model types, the MobileViT network performed the best with an average accuracy of 98.93%, followed by MobileNetV3 in second place with 98.69%, ResNet18 in third place with 96.41%, and VGG16 in last place with 95.85%.

By organizing Table 2 according to the type of goat appearance, the average accuracy of the corresponding models for each type of appearance is shown in Table 3. It can be seen that the side body appearance had the best performance, with an average accuracy of 99.1%, followed by the back body appearance, with an average accuracy of 97.92%; the front face appearance was ranked third, with an average accuracy of 97.55%; the right face appearance was ranked fourth, with an average accuracy of 96.41%; and the left face appearance had the worst performance, with an average accuracy of 96.37%. Combining Table 2 and Table 3 reveals that when using the same base models, the accuracy of the side body appearance corresponding models was the highest. The accuracy of the back body appearance corresponding models was ranked second, while the accuracy of the front face appearance corresponding models was ranked third, and it was not easy to distinguish between the accuracy of the two side face appearance corresponding models. Even in the worst performing VGG16 series models, the accuracy and F1 score of the side body view appearance model (VGG16-S) also reached 98.52% and 98.51%, respectively, being 4.63% and 4.60% higher than that of the left face view appearance model (VGG16-L), which was the worst test. In conclusion, the side body view appearance series of models performed best in the goat identification task for a single viewpoint of the appearance.

### 3.3. Multiple Appearance Fusion Model

The performance of the models using two types of appearance fusion on the test set is shown in Table 4. The Top-1 Acc indicators of all models exceeded 99.1%, performing better than single appearance models. The recognition accuracy of multiple models reached 100%, achieving zero-error identification performance. Among them, in MobileNetV3, the model with the worst performance achieved accuracy and an F1 score of 99.72% after fusing right face and back body appearances (MobileNetV3-R-B). In comparison, other fusion models combining dual-view appearances achieved accuracies and F1 scores of over 99.91%. Compared to identity recognition using only the right face appearance model (MobileNetV3-R), the accuracy and F1 score were increased by at least 1.85%. In VGG16, which performed the worst in single-view appearance identity recognition, the recognition performance significantly improved after using dual-view appearance fusion. The models combining the front face appearance with (left or right) side face appearance (VGG16-F-L or VGG16-F-R) achieved accuracy improvements of 3.61% and 3.7%, respectively, compared to using only the front face appearance model (VGG16-F), with corresponding F1 score improvements of 3.6% and 3.7%. The model fusing left and right side face appearance (VGG16-L-R) achieved accuracy improvements of 5.46% and 5.09%, respectively, compared to using only the single side face appearance model (VGG16-L or VGG16-R), with corresponding F1 score improvements of 5.44% and 5.07%. For ResNet18, which showed mediocre performance in single-view appearance identity recognition, the accuracy and F1 score of the worst-performing fusion model reached 99.54%. MobileViT, which performed optimally in single-view appearance identity recognition, showed comprehensive improvements in recognition performance after dual-view appearance fusion, with three fusion models achieving accuracies and F1 scores of 99.91% and the rest reaching 100%.

For the models in Table 4, after organizing them according to the different combinations of goats’ appearances, the average accuracy for identifying goat individual identities under various combinations of two appearances is shown in Table 5. It can be observed that when fusing two appearances, the performance was best when combining the side body and back body appearances (S-B), with an average accuracy of 99.96%. In contrast, the fusion of the right face and back body appearances (R-B) performed the worst, with an average accuracy of 99.74%.

The performances of goat identity recognition models with three, four, and five appearance fusion on the test set are shown in Table 6, Table 7, and Table 8, respectively. The Top-1 Acc of models obtained after three types of appearance fusions almost all reached 100%. In comparison, the Top-1 Acc of models obtained after four and five types of appearance fusions were all 100%, achieving comprehensive zero-error recognition of the identities of all 54 goats.

In summary, the best combination of side and back appearance was achieved when two appearance images were used, and its average accuracy was 99.96; when three or more appearances were used, the average accuracy was almost 100%.

### 3.4. Calculated Load for Each Network Model

In this study, the computational effort of the single appearance recognition model was calculated based on PyTorch, and the results are shown in Table 9. Since the additional computation required to perform the multi-view appearance fusion step is very small, the computation effort of the multi-view appearance identity recognition model is approximately equal to the multiple of each single appearance identity recognition model. For example, the model computation effort for a three-view image is three times the computation effort for a single-view model. In practical applications, whether it is a single-view appearance identity recognition model or a multi-view fusion identity recognition model, the actual computational effort of the model is not only related to the complexity of the model but also affected to a greater extent by the number of recognized images. In other words, minimizing the number of external images of goats used minimizes the computational load of the model.

## 4. Analysis and Discussion

This study collected five types of appearance images of the left face, front face, right face, back body, and side body of 54 Wanlin white goats. Based on four base network models, MobileNetV3, MobileViT, ResNet18, and VGG16, one, two, three, four, and five types of goat appearance were considered to establish the goat individual identity recognition model. The results show that the goat side body appearance image had the most powerful ability to recognize the goat’s identity in single appearances; the fusion of multiple appearances significantly improved the accuracy of the goat’s identity recognition. When the number of appearance types was greater than or equal to three, the accuracy of the fusion model on the individual identity recognition was almost 100%, and it achieved nearly zero error identity.

### 4.1. Single Appearance Recognition Model

The reasons for the differences in recognizing individual identities by different types of goats’ appearances were analyzed as follows. As shown in Table 2, the performance of the models constructed based on the four base networks consistently indicates that using the side body appearance had the highest accuracy in all cases. The back body appearance was the next most accurate, and the front face was higher than the two side face appearances by a lower advantage. In contrast, the left and right sides of the face showed a minor difference in recognition accuracy. This was because the side body view appearance was more stable than the other view appearances, being able to accurately display the goat’s physical features, body contours, body structure, and proportions. It is also less susceptible to conditions such as light and goat activity and thus helps improve recognition accuracy. The appearance of the back body view was slightly worse, although the goat’s back body appearance was also more stable. The back body view was more sensitive to the interference of lighting factors than the other views under natural lighting conditions. The heterogeneous lighting may lead to problems such as shadows, reflections, and highlights, affecting the image’s quality and the recognition’s accuracy. Although the front face view appearance was considered the most intuitive and familiar angle in most cases, compared to other animals, the Wanlin white goats have a higher nose and a more three-dimensional face, and the goat’s head can be moving at any time, which results in a lack of sufficient appearance information in the captured single-view front face image; moreover, there are the high degree of similarity of the faces and the lack of salient differences in the facial features, which results in a lower recognition accuracy compared to the side body and back body. Because the side face features of goats are relatively symmetrical, the difference between the facial features of the left and right faces is insignificant, so the model has a minor difference in accuracy in identity recognition. A slightly lower accuracy of the side face relative to the front face appearance may be because the front face appearance contains more comprehensive facial feature information.

**Figure 11:**
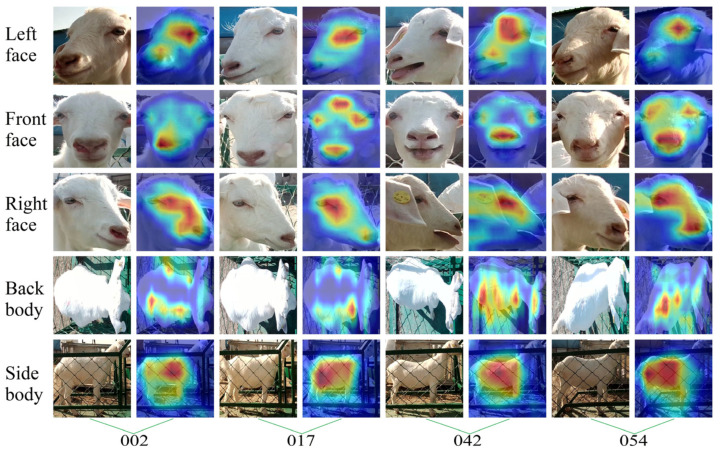
Visual examples of goat identity recognition based on the MobileNetV3 network model. The column is the ID number corresponding to the selected goats, and the row shows the different appearance types of the goats.

**Figure 12:**
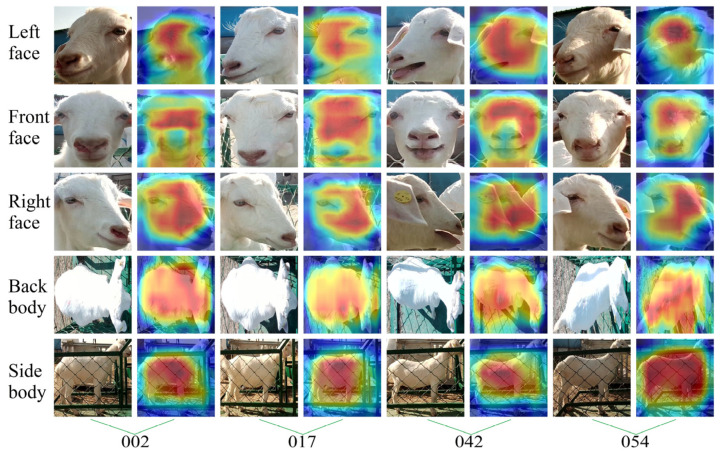
Visual examples of goat identity recognition based on the MobileViT network model. The column is the ID number corresponding to the selected goats, and the row shows the different appearance types of the goats.

**Figure 13:**
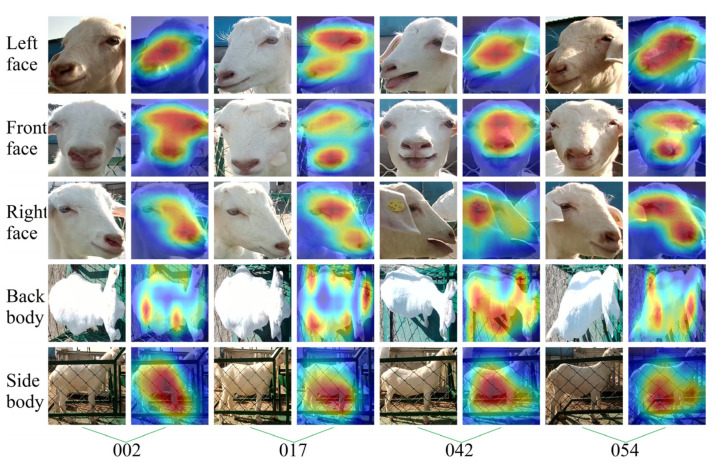
Visual examples of goat identity recognition based on the ResNet18 network model. The column is the ID number corresponding to the selected goats, and the row shows the different appearance types of the goats.

**Figure 14:**
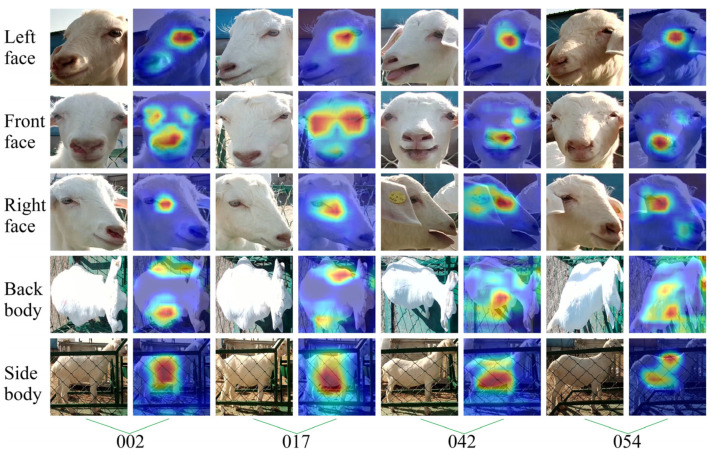
Visual examples of goat identity recognition based on the VGG16 network model. The column is the ID number corresponding to the selected goats, and the row shows the different appearance types of the goats.

The attention to goat appearance in each goat’s identification model was analyzed to further reveal the parts of appearance features extracted by different models for different appearance images of goats when recognizing the individual identity of goats. This study randomly selected four goats (ID: 0002, 0017, 0042, 0054, respectively), using the Grad-CAM++ visualization model on their identity recognition process, as shown in Figure 11, Figure 12, Figure 13 and Figure 14.

By observing Figure 11, it can be noted that MobileNetV3-L, MobileNetV3-F, and MobileNetV3-R accurately focused on the facial area of the goat, especially paying attention to the inner corner of the eyes and the frizz of the hair. MobileNetV3-S accurately paid attention to the side of the goat’s body, while MobileNetV3-B accurately focused on both sides of the goat’s back body. The visualization features at the spine were not reflected in the figure, which may have been due to the top-down view of the back body being affected by bright light, resulting in varying levels of exposure in different areas of the back body.

Observation of Figure 12 shows that, relative to the visualizations performed by the other networks, MobileViT had a broader range of attention on the image. MobileViT-L, MobileViT-F, and MobileViT-R can accurately focus attention on the position of the goat’s face, which is more prominent, especially on the cheeks and the bridge of the nose. MobileViT-S can accurately focus on the side of the goat’s body. MobileViT-B can accurately focus on the entire back body of the goat. The reason is that MobileViT, based on a hybrid approach combining CNN and Vision Transformer, can realize the effective extraction of local and global features, which can capture the object features more comprehensively, reduce the effect of lighting, and highlight the perception of global information.

By observing Figure 13, it can be seen that ResNet18-L, ResNet18-F, and ResNet18-R can accurately focus attention on the location of the goat’s face, which is especially obvious in the region near the bridge of the nose with the ear labels. ResNet18-S can accurately focus on the location of the side of the goat’s body, which is especially obvious in the abdomen with the front legs. ResNet18-B accurately focuses on the non-exposed areas of the goat’s back body, which again confirms that top-view images are susceptible to image exposure when using CNNs for feature extraction.

Observing Figure 14 shows that VGG16-L, VGG16-F, and VGG16-R can accurately focus attention on the location of the goat face, especially more evident at the corner of the inner eye and the tip of the nose. VGG16-S can accurately focus on the location of the side of the goat’s body, especially more evident at the abdomen and the dorsal arch. VGG16-B focuses on the non-exposed region of the goat’s back body, re-emphasizing that top-view images are susceptible to image exposure when using CNNs for feature extraction.

Taken together, the above analysis shows that for three types of facial appearance, the degree of visualization of the activation map features at the eyes, mouth, and nose is small, and the model relies more on the comprehensive texture of the hair and the facial structural features to carry out the identity recognition. As for the back body appearance, because the goat’s body is white, the back body appearance is easily affected by light, so most models focus on the two sides of the spine of the back body with clear hair texture for identification. As for the sided body appearance, most models focused on the lateral body part between the front and back body limbs, relying on the integrated hair texture and spinal and abdominal structural features for stable identification. In conclusion, different goat appearances have different degrees of contribution to the identification of goats, among which the side body view appearance is the most stable, and the MobileViT network is the strongest in extracting feature information.

### 4.2. Multiple Appearance Fusion Model

The reasons for accurately recognizing the individual identity of the goat using multiple appearances are analyzed as follows. As shown in Table 4, Table 6, Table 7 and Table 8, using any two-view appearance fusion can achieve high identification accuracy; most networks can achieve 100% identification accuracy when using three-view appearance fusions; any network was able to identify 100% of the goat when using more than four view appearance fusions. The reason is that the prediction result of each viewpoint model is affected by the weighted average fusion, the probability of the prediction result for the same identity increases, and the likelihood of the prediction result for different identities decreases with the increased number of appearance types. This increases the gap between correct and incorrect predictions and makes the prediction results more accurate. Compared to other viewpoint appearance fusions, most models that use side body viewpoint fusion with any other viewpoint appearance perform better. The reason is that the side body appearance performs best in single recognition models, and thus its corresponding weight is the largest and more influential in the final decision results.

### 4.3. Strategies for Goat Individual Identification in Practical Applications

Identifying individual identities using multiple goat appearances has the advantage of zero error. However, as the types of appearance images used increase, the computational cost also increases. Designing an optimal cost-effective recognition strategy is very necessary for practical applications.

**Figure 15:**
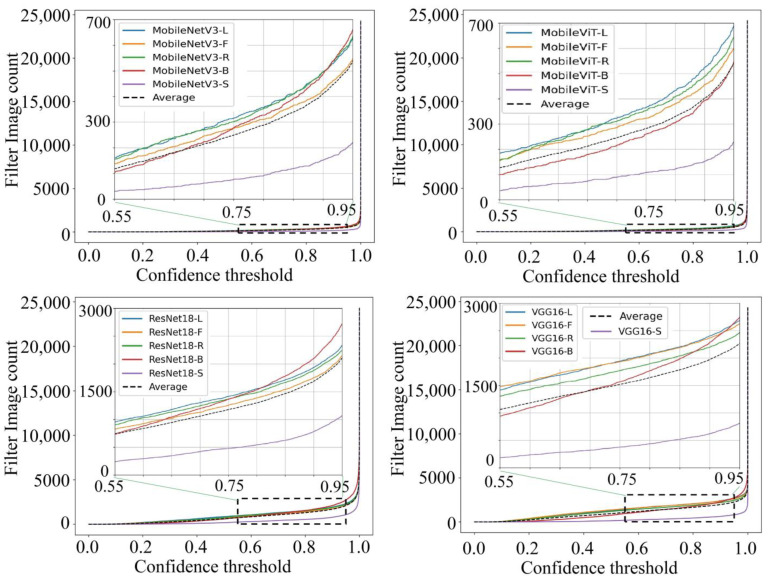
The curve graph of the relationship between the Top-1 confidence threshold for goat identity recognition and the number of images in the training set sample that did not reach the specified threshold, based on the goat single appearance recognition models of MobileNetV3, MobileViT, ResNet18, and VGG16. Here, Model-L: model trained based on goats’ left side face images; Model-F: model trained based on goats’ front face images; Model-R: model trained based on goats’ right side face images; Model-B: model trained based on goats’ back body images; Model-S: model trained based on goats’ side body images. Average: average of 5 types of appearance.

The Top-1 confidence thresholds of the 20 single appearance recognition models for the training set to predict goat identity were statistically analyzed against the number of images that did not reach the specified thresholds, as shown in Figure 15. It can be seen that in MobileNetV3 and MobileViT single appearance recognition models using training image prediction, the number of images with Top-1 confidence thresholds lower than 0.9 accounted for less than 2% of the total number of images, and lower than 0.6 accounted for less than 1% on average. In ResNet18 and VGG16 single appearance recognition models using training image prediction, the average number of images with a Top-1 confidence threshold lower than 0.9 was less than 8% of the total number, and lower than 0.6 was less than 5%. Most training images were predicted to have Top-1 confidence thresholds close to 1 for all the goats’ single appearance recognition models. This indicates that the vast majority of images in the training set were predicted with high confidence. This suggests that by setting a reasonable Top-1 confidence threshold, the performance of the recognition model using a single appearance can be improved with the assurance of filtering out a smaller number of images under accuracy.

Combined with Table 10, the recognition accuracies of the goat single appearance recognition models under different Top-1 confidence thresholds when using the test set data for identity recognition are shown. The results show that after applying the Top-1 confidence threshold to filter the images with a low threshold, the recognition accuracy of the model increased significantly with the increase in the threshold. When the Top-1 confidence threshold was set to 0.9, the Top-1 Acc was over 99.6%. This indicates that most recognition errors were in the low threshold case, and the lower the threshold, the higher the proportion of incorrectly recognized images.

Based on the above results, for the single-camera condition, we propose to implement the following strategy to recognize the identity of a goat: when one goat appearance image needs to be identified, if the Top-1 confidence threshold of the model prediction is greater than or equal to 0.9, the result is considered to be trustworthy, and the result is outputted. If the Top-1 confidence threshold is between 0.7 and 0.9, reshoot a new goat appearance image, combine the new recognition results of the reshooted appearance image, and determine the prediction results using the voting method [19]. Specifically, in the case of the lowest number of images of captured goat appearance, the prediction result with the same output as the majority of the images is the voting result for the identity of the goat. When the Top-1 confidence threshold is less than 0.7, we consider that the prediction result of this image is not worthy of reference, and we should then obtain new goat appearance images to recognize it again.

Strategies for recognizing the identity of individual goats under multi-camera conditions: As mentioned above, we can improve the recognition performance by using strategies in conditions with only one camera device. However, as the set Top-1 confidence threshold increases, it may be tricky to capture images that can recognize the identity of a goat under only one camera, resulting in a long time for goat individual identification. Luckily, the multi-view camera device overcomes the shortcomings of the single-camera device. It can simultaneously capture the goat’s appearance from different angles, significantly reducing the time needed to obtain the ideal appearance images. In practice, for multi-camera conditions, we propose implementing the following strategy to identify goats: the side body appearance is first recognized using the goat single appearance recognition model; if the Top-1 confidence threshold predicted by the model is greater than or equal to 0.9, the result is considered to be trustworthy. Otherwise, if the Top-1 confidence threshold is less than 0.9, add one goat back body appearance image captured through the overhead camera and use the side body and back body images to output the Top-1 confidence through the weighted fusion model. If the Top-1 confidence threshold is greater than or equal to 0.9 at this time, the result is considered to be trustworthy. Suppose the Top-1 confidence threshold is less than 0.9. Add one goat front face appearance image captured by the front face camera, and use side body, back body, and front face images through the weighted fusion model recognition results. Specifically, fusion utilizing the appearance of three viewpoints achieves 100% identity recognition accuracy on most networks. The hybrid structure of series and parallel connection is used to obtain the appearance feature information of goats from multiple sources on the optimal computational path, which saves computational resources and greatly improves the individual recognition ability of goats. Of course, we can also adaptively select other multi-view appearances that are easier to obtain to adapt to the actual application scenario by introducing on-demand in order to realize the individual identity of the goat to ensure the recognition accuracy while saving computational volume and recognition time.

### 4.4. Comparison with Reported Studies and Subsequent Research Plans

Previous studies [5,16,19] have directly pointed out that using the front face appearance images to recognize the individual identity of sheep is prone to higher recognition accuracy. However, this conclusion does not take into account the side and top views of the sheep’s body. This study used different typical networks (to improve the generalizability of the study) to construct network models for the five views of the appearance of goats, as well as through a large number of experiments, revealing the ability of different views of appearance image information for goat identity recognition, proving that side and back body images are better than front-face images.

Previous studies have favored using only front-face images to identify the identity of goats, which is challenging to implement in complex environments where the sheep’s head posture is dynamically changing and the desirable view is difficult to obtain [18]. Moreover, due to limited information on the single-view front-face image [14], it is difficult to distinguish between goats of the same breed with highly similar appearances. Multi-view appearance fusion reduces the impact of goat posture and has more information about the sheep’s appearance. Strategies for using sheep appearance information on demand ensure recognition accuracy while saving computation and recognition time.

In contrast to previous studies on network improvement for recognition [8,20,21,28], this study is not limited to improving the accuracy and speed of goat individual identity recognition from a single viewpoint appearance by improving the network structure. It aims to utilize the contribution of different appearance views to goat individual identity recognition. Decision fusion is made by averaging more appearance information features embedded in multi-views by weighting the contributions to obtain a 100% accurate recognition strategy for goat individual identity using the minimum amount of computation.

Unfortunately, due to the limitations of experimental conditions and resources, this study achieved good results only on Wanlin white goats, whose appearance is extremely similar. It was difficult to obtain sufficient multi-view appearance image data of other breeds of goats or sheep to conduct more extensive experimental validation. There is still some work here that deserves further improvement. During the acquisition of front facial images, passive waiting for the ideal sheep head posture resulted in acquiring a much lower number of sheep face images than the other views. The next step of the study should be to automatically adjust the camera’s position according to the sheep’s head posture to capture the ideal appearance image. This transformation from manual acquisition to automatic capture also complies with demand in practical applications.

## 5. Conclusions

Through a self-constructed multi-view goat appearance image acquisition platform, this study collected five types of appearance from 54 Wanlin white goats and constructed a series of goat identity recognition models utilizing one, two, three, four, and five types of appearance based on four typical backbone network models. The results show that among the five appearance images, the side body image had the strongest ability to recognize goat identity, the back body image was the second best, the front face image was slightly better than the two side face images, and the difference between the left and right side faces was small. When only one kind of goat appearance image was used, the combination of side body image and MobileViT was the best, with an accuracy of 99.63%; under identity recognition based on multi-source image appearance fusion, all recognition models after outlook fusion of two viewpoints generally outperformed single viewpoint appearance identity recognition models in recognizing the identity of individual goats; when three and more kind of goat appearance images were utilized for fusion, any of the four models were capable of identifying the identity of an individual goat with 100%.

In practical applications, increasing the images of sheep appearance sequentially according to the confidence level can balance the recognition accuracy, computation, and time of the model. For example, starting from using the sheep side body appearance image, when the Top-1 confidence threshold of the model was between 0.7 and 0.9, the back body image, the front face image, and the side face image were added sequentially until the confidence threshold of the model output was greater than 0.9. The hybrid structure of series and parallel connection is used to obtain the appearance feature information of goats from multiple sources on the optimal computational path, which saves computational resources and greatly improves the individual recognition ability of goats. The five appearance images of the sheep in this study were obtained by manual processing and should be further automated for acquisition in future studies.

The results revealed in this study on the ability of the five appearances images of goats and their combinations to recognize the individual identity of goats will provide a solid theoretical basis for the development of a more practical individual identity recognition system for goats, which will help to promote the process of precision in goat husbandry.

## Figures and Tables

**Table 1:** Localization results of the YOLOv8n object detection model.

Model	Precision	Recall	mAP@0.5	mAP@0.5–0.95
YOLOv8n	99.4	99.5	99.4	96.8

Note: AP refers to average precision; mAP@0.5 refers to the mean AP of all categories when the IoU threshold is set to 0.5; mAP@0.5–0.95(%) indicates the average mAP over different IoU thresholds from 0.5 to 0.95 in steps of 0.05.

**Table 2:** Recognition results of the single-view appearance identity recognition model.

Model	Top-1 Acc	Top-5 Acc	Mean Precision	Mean Recall	Mean F1 Score
MobileNetV3-L	98.15	99.35	98.22	98.15	98.13
MobileNetV3-F	98.8	99.72	98.86	98.8	98.8
MobileNetV3-R	97.87	99.44	98	97.87	97.87
MobileNetV3-B	99.07	99.91	99.12	99.07	99.08
MobileNetV3-S	99.54	100	99.56	99.54	99.54
MobileViT-L	98.52	99.44	98.59	98.52	98.52
MobileViT-F	98.98	99.72	99.02	98.98	98.98
MobileViT-R	98.24	99.54	98.33	98.24	98.24
MobileViT-B	99.26	99.91	99.3	99.26	99.26
MobileViT-S	99.63	100	99.66	99.63	99.63
ResNet18-L	94.91	98.06	95.13	94.91	94.89
ResNet18-F	96.48	99.07	96.61	96.48	96.46
ResNet18-R	95.28	98.89	95.49	95.28	95.28
ResNet18-B	96.67	99.17	96.77	96.67	96.67
ResNet18-S	98.7	99.91	98.77	98.7	98.7
VGG16-L	93.89	97.78	94.14	93.89	93.91
VGG16-F	95.93	98.43	96.07	95.93	95.93
VGG16-R	94.26	97.59	94.5	94.26	94.28
VGG16-B	96.67	99.07	96.78	96.67	96.66
VGG16-S	98.52	99.91	98.59	98.52	98.51

Note: Top-1 Acc is the probability that the most likely category predicted by the model matches the actual label; Top-5 Acc is the probability that one of the top five most likely categories predicted by the model contains the actual label.

**Table 3:** The average performance of individual identity recognition in goats of each appearance.

Appearance Type	Top-1 Acc	Top-5 Acc	Mean Precision	Mean Recall	Mean F1 Score
L	96.37	98.66	96.52	96.37	96.36
F	97.55	99.24	97.64	97.55	97.54
R	96.41	98.87	96.58	96.41	96.42
B	97.92	99.52	97.99	97.92	97.92
S	99.1	99.96	99.15	99.1	99.1

Note: Top-1 Acc is the probability that the most likely category predicted by the model matches the actual label; Top-5 Acc is the probability that one of the top five most likely categories predicted by the model contains the actual label.

**Table 4:** Recognition results of the dual-view appearance identity recognition models.

Model	Top-1 Acc	Top-5 Acc	Mean Precision	Mean Recall	Mean F1 Score
MobileNetV3-S-F	100	100	100	100	100
MobileNetV3-S-R	99.91	100	99.91	99.91	99.91
MobileNetV3-S-L	99.91	100	99.91	99.91	99.91
MobileNetV3-S-B	100	100	100	100	100
MobileNetV3-F-R	100	100	100	100	100
MobileNetV3-F-L	99.91	99.91	99.91	99.91	99.91
MobileNetV3-F-B	100	100	100	100	100
MobileNetV3-L-R	99.91	100	99.91	99.91	99.91
MobileNetV3-R-B	99.72	100	99.74	99.72	99.72
MobileNetV3-L-B	99.91	100	99.91	99.91	99.91
MobileViT-S-F	100	100	100	100	100
MobileViT-S-R	99.91	100	99.91	99.91	99.91
MobileViT-S-L	100	100	100	100	100
MobileViT-S-B	100	100	100	100	100
MobileViT-F-R	99.91	100	99.91	99.91	99.91
MobileViT-F-L	99.91	100	99.91	99.91	99.91
MobileViT-F-B	100	100	100	100	100
MobileViT-L-R	100	100	100	100	100
MobileViT-R-B	100	100	100	100	100
MobileViT-L-B	100	100	100	100	100
ResNet18-S-F	99.91	100	99.91	99.91	99.91
ResNet18-S-R	99.91	100	99.91	99.91	99.91
ResNet18-S-L	99.91	99.91	99.91	99.91	99.91
ResNet18-S-B	99.91	100	99.91	99.91	99.91
ResNet18-F-R	99.72	99.91	99.74	99.72	99.72
ResNet18-F-L	99.81	99.91	99.82	99.81	99.81
ResNet18-F-B	99.72	100	99.73	99.72	99.72
ResNet18-L-R	99.72	100	99.74	99.72	99.72
ResNet18-R-B	99.81	100	99.82	99.81	99.81
ResNet18-L-B	99.54	100	99.56	99.54	99.54
VGG16-S-F	99.72	100	99.74	99.72	99.72
VGG16-S-R	99.72	100	99.74	99.72	99.72
VGG16-S-L	99.81	100	99.82	99.81	99.81
VGG16-S-B	99.91	100	99.91	99.91	99.91
VGG16-F-R	99.63	100	99.66	99.63	99.63
VGG16-F-L	99.54	99.91	99.56	99.54	99.53
VGG16-F-B	99.72	100	99.74	99.72	99.72
VGG16-L-R	99.35	100	99.38	99.35	99.35
VGG16-R-B	99.44	99.72	99.47	99.44	99.44
VGG16-L-B	99.63	100	99.65	99.63	99.63

Note: Top-1 Acc is the probability that the most likely category predicted by the model matches the actual label; Top-5 Acc is the probability that one of the top five most likely categories predicted by the model contains the actual label.

**Table 5:** The performance of two appearance fusions in goat individual identity recognition.

Appearance Fusion	Top-1 Acc	Top-5 Acc	Mean Precision	Mean Recall	Mean F1 Score
S-F	99.91	100	99.91	99.91	99.91
S-R	99.86	100	99.87	99.86	99.86
S-L	99.89	99.98	99.89	99.89	99.89
S-B	99.96	100	99.96	99.96	99.96
F-R	99.84	99.98	99.85	99.84	99.84
F-L	99.79	99.93	99.8	99.79	99.79
F-B	99.86	100	99.87	99.86	99.86
L-R	99.75	100	99.76	99.75	99.75
R-B	99.74	99.93	99.76	99.75	99.75
L-B	99.77	100	99.78	99.77	99.77

Note: Top-1 Acc is the probability that the most likely category predicted by the model matches the actual label; Top-5 Acc is the probability that one of the top five most likely categories predicted by the model contains the actual label.

**Table 6:** Recognition results of the three-view appearance identity recognition model.

Model	Top-1 Acc	Top-5 Acc	Mean Precision	Mean Recall	Mean F1 Score
MobileNetV3-All	100	100	100	100	100
MobileViT-All	100	100	100	100	100
ResNet18-All	100	100	100	100	100
VGG16-Others	100	100	100	100	100
VGG16-F-R-L	99.91	100	99.91	99.91	99.91
VGG16-R-L-B	99.91	100	99.91	99.91	99.91

Note: MobileNetV3-All denotes the set of all three-view appearance fusion models of goats in MobileNetV3; MobileViT-All denotes the set of all three-view appearance fusion models of goats in MobileViT; ResNet18-All denotes the set of all three-view appearance fusion models of goats in ResNet18; VGG16-Others denotes the set of all goat three-view appearance fusion models in VGG16 removing the VGG16-F-R-L model and the VGG16-R-L-B model; Top-1 Acc is the probability that the most likely category predicted by the model matches the actual label; Top-5 Acc is the probability that one of the top five most likely categories predicted by the model contains the actual label.

**Table 7:** Recognition results of the four-view appearance identity recognition model.

Model	Top-1 Acc	Top-5 Acc	Mean Precision	Mean Recall	Mean F1 Score
MobileNetV3-All	100	100	100	100	100
MobileViT-All	100	100	100	100	100
ResNet18-All	100	100	100	100	100
VGG16-All	100	100	100	100	100

Note: MobileNetV3-All denotes the set of all four-view appearance fusion models of goats in Mo-bileNetV3; MobileViT-All denotes the set of all four-view appearance fusion models of goats in MobileViT; ResNet18-All denotes the set of all four-view appearance fusion models of goats in ResNet18; VGG16-All denotes the set of all four-view appearance fusion models of goats in VGG16; Top-1 Acc is the probability that the most likely category predicted by the model matches the actual label; Top-5 Acc is the probability that one of the top five most likely categories predicted by the model contains the actual label.

**Table 8:** Recognition results of the five-view appearance identity recognition models.

Model	Top-1 Acc	Top-5 Acc	Mean Precision	Mean Recall	Mean F1 Score
MobileNetV3-L-F-R-B-S	100	100	100	100	100
MobileViT-L-F-R-B-S	100	100	100	100	100
ResNet18-L-F-R-B-S	100	100	100	100	100
VGG16-L-F-R-B-S	100	100	100	100	100

Note: Top-1 Acc is the probability that the most likely category predicted by the model matches the actual label; Top-5 Acc is the probability that one of the top five most likely categories predicted by the model contains the actual label.

**Table 9:** Computational effort per base network model.

Metrics	MobileNetV3	MobileViT	ResNet18	VGG16
FLOPs/G	0.06	1.44	1.82	15.5
Params/M	1.57	4.97	11.2	134.48

Note: FLOPs denote the number of floating point operations, understood as the amount of computation, which is used to measure the computational complexity of the model; Params denotes the number of parameters of the model.

**Table 10:** Recognition results of the single-view appearance identity recognition models under different Top-1 confidence thresholds.

Model	Top-1 @0	Top-1 Acc@0.6	Top-1 Acc@0.7	Top-1 Acc@0.8	Top-1 Acc@0.9
MobileNetV3-L	98.15	99.15	99.24	99.62	99.71
MobileNetV3-F	98.8	99.25	99.25	99.64	99.9
MobileNetV3-R	97.87	99.15	99.24	99.81	99.9
MobileNetV3-B	99.07	99.35	99.44	99.53	99.91
MobileNetV3-S	99.54	99.63	99.63	99.63	99.81
MobileViT-L	98.52	99.43	99.71	99.81	99.81
MobileViT-F	98.98	99.53	99.72	99.81	99.91
MobileViT-R	98.24	99.06	99.52	99.71	99.81
MobileViT-B	99.26	99.53	99.72	99.72	99.81
MobileViT-S	99.63	99.81	99.81	99.81	99.91
ResNet18-L	94.91	98.12	98.70	99.29	99.79
ResNet18-F	96.48	98.56	98.83	99.21	99.6
ResNet18-R	95.28	98.53	99.2	99.4	99.79
ResNet18-B	96.67	98.66	99.22	99.41	99.8
ResNet18-S	98.7	99.25	99.43	99.81	99.91
VGG16-L	93.89	98.51	98.6	98.88	99.38
VGG16-F	95.93	98.64	98.91	99.4	99.8
VGG16-R	94.26	98.22	98.8	99.39	99.79
VGG16-B	96.67	98.56	99.22	99.51	99.81
VGG16-S	98.52	99.16	99.72	99.81	99.81

Note: “@0” refers to no threshold; “@0.6” refers to a threshold of 0.6; “@0.7” refers to a threshold of 0.7; “@0.8” refers to a threshold of 0.8; “@0.9” refers to a threshold of 0.9.

## Data Availability

All relevant data are included in the article.
